# Zero-Inflated Time Series Clustering Via Ensemble Thick-Pen Transform

**DOI:** 10.1007/s00357-023-09437-z

**Published:** 2023-06-12

**Authors:** Minji Kim, Hee-Seok Oh, Yaeji Lim

**Affiliations:** 1 Department of Statistics and Operations Research, University of North Carolina at Chapel Hill, North Carolina, USA; 2 Department of Statistics, Seoul National University, 08826 Seoul, Korea; 3 Department of Applied Statistics, Chung-Ang University, 48513 Seoul, Korea

**Keywords:** Clustering, Multiscale method, Newly confirmed COVID-19 case data, Step count data, Thick-pen transform, Zero-inflated time series data

## Abstract

This study develops a new clustering method for high-dimensional zero-inflated time series data. The proposed method is based on thick-pen transform (TPT), in which the basic idea is to draw along the data with a pen of a given thickness. Since TPT is a multi-scale visualization technique, it provides some information on the temporal tendency of neighborhood values. We introduce a modified TPT, termed ‘ensemble TPT (e-TPT)’, to enhance the temporal resolution of zero-inflated time series data that is crucial for clustering them efficiently. Furthermore, this study defines a modified similarity measure for zero-inflated time series data considering e-TPT and proposes an efficient iterative clustering algorithm suitable for the proposed measure. Finally, the effectiveness of the proposed method is demonstrated by simulation experiments and two real datasets: step count data and newly confirmed COVID-19 case data.

## Introduction

Clustering is a popular unsupervised machine learning technique for identifying patterns and groupings in data, which has been widely used in many domains, including biology, finance, and image processing. However, many real-world datasets, especially those in healthcare, finance, and environmental monitoring, often exhibit zero inflation, which refers to excessive zeros in the data. This characteristic poses significant challenges to traditional clustering algorithms, which assume that the data points follow a specific distribution or pattern. In particular, zero-inflated time series data are prevalent in many domains, such as disease surveillance and financial transaction analyses. For instance, in epidemiology, the counts of infectious diseases are often zero-inflated due to under-reporting, misclassification, and other factors. Various methods have been proposed to address the challenges of clustering zero-inflated time series data, including a zero-inflated Gaussian mixture model (Zhang et al., 2020), a zero-inflated Poisson mixture model (Lim et al., 2014), and a zero-inflated negative binomial mixture model (Yau et al., 2003). These methods aim to model the zero-inflation by adding extra parameters or components to the mixture model and effectively cluster zero-inflated time series data.

In this study, we propose a new clustering method without specific model assumptions that can be applied to various structures of zero-inflated time series data. Selecting an appropriate distance (similarity) measure in time series data clustering is essential. Thus, we propose a similarity measure suitable for zero-inflated time series data inspired by the thick-pen transform (TPT) by Fryzlewicz & Oh (2011). The TPT is a novel way of visualizing time series data at multiple scales using a range of pens with various thicknesses. To improve the temporal resolution of zero-inflated time series data, which is crucial for efficient clustering, we introduce two modifications: the ensemble TPT (e-TPT) and a modified similarity measure called TPMA$_0$. These approaches have the advantage of overriding some original properties of the TPT, capturing time series trends of neighboring data points, and reflecting the multi-scale information of the data. Then, we present a clustering algorithm based on the proposed similarity measure.

**Fig. 1:**
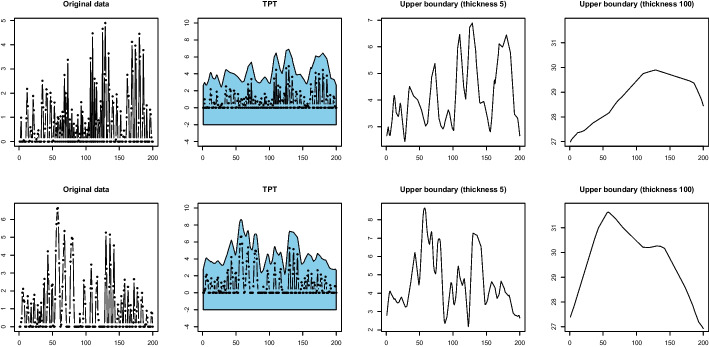
From left to right, two simulated zero-inflated time series, their e-TPT with a square pen with a thickness of 5, and upper boundaries obtained from the e-TPT with thicknesses of 5 and 100

The primary rationale of the proposed method is that e-TPT can effectively manage the issue of excessive zeros in zero-inflated time series data. To demonstrate this, we present two zero-inflated time series in Fig. 1, where the proportions of zero observations are 0.495 and 0.480. We apply e-TPT with a square pen, as explained in Section 2.1, and obtain the upper boundary of the pen. The lower boundary of the e-TPT for zero-inflated time series data rarely fluctuates; thus, we only consider the upper boundary of the pen. The upper boundary from the pen with a thickness of 100 manifests the global trend of the data from the two time series, and the two series are distinguished. Moreover, there is no zero observation in the upper boundary of the e-TPT, indicating that a simple clustering method may work well without considering the problem of exceeding zero.

**Fig. 2:**
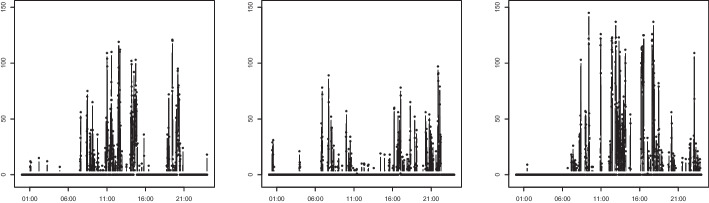
Step count data for three different days

This study is motivated by two real-world time series. The first comprises data on the number of steps recorded from wearable devices. Figure 2 depicts the step data recorded for three days. As expected, zero values occur frequently, and daily activity patterns are observed. A proper clustering of step data can provide rich information about physical activities and can be further used for personal healthcare services.

**Fig. 3:**
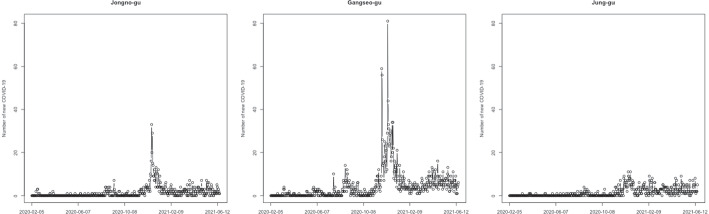
Newly confirmed COVID-19 cases per day in three districts of Seoul from February 5, 2020 to June 18, 2021

We consider newly confirmed coronavirus disease 2019 (COVID-19) cases per day in Seoul, Korea, as the second time series dataset. South Korea had its first confirmed COVID-19 case in January 2020. As of February 2022, the cumulative number of confirmed cases was more than 2,665,000. Figure 3 illustrates the number of new COVID-19 cases per day in three districts in Seoul from February 5, 2020, to June 18, 2021. Before November 2020, few new cases of COVID-19 were confirmed in all three districts, but the number of confirmed cases suddenly increased in the winter of 2020. The days with zero confirmed cases are 51.6%, 31.6%, and 50%, respectively. This data analysis aims to observe the time series patterns of confirmed cases that vary from district to district and cluster the 25 districts in Seoul based on the patterns of confirmed COVID-19 cases per day. Recently, many COVID-19-related studies have been conducted, and the number of deaths or confirmed cases is modeled using zero-inflated time series models. For example, Tawiah et al. (2021) analyzed the trend of a daily count of COVID-19 deaths in Ghana using a zero-inflated Poisson autoregressive model and a zero-inflated negative binomial autoregressive model.

The remainder of this paper is organized as follows. Section 2 introduces an e-TPT and proposes a new similarity measure based on the e-TPT. In addition, the proposed clustering method and its practical algorithm are presented. In Section 3, a simulation study is conducted to evaluate the empirical performance of the proposed method. Next, Section 4 discusses the real-data analysis with two real datasets: step-count data and newly confirmed COVID-19 cases data. The concluding remarks are provided in Section 5.

## Proposed Clustering Procedure

### Ensemble Thick-Pen Transform

The TPT is based on the idea of drawing along time series data points with a pen with a shape and thickness. We let $\mathcal {J} = \{\tau _j >0 : j=1,\dots ,|\mathcal {J}| \}$ denote a set of thickness parameters. The TPT of a real-valued univariate process $\{X(t)\}_{t=1}^T$ is defined as the following sequence of boundary pairs:

$$ \textbf{TP}_{\mathcal {J}}(X(t)) = \{ (L_{\tau _j}(X(t)), U_{\tau _j}(X(t))) \}_{j=1,\dots ,|\mathcal {J}|}, $$

where $L_{\tau _{j}}(X(t))$ and $U_{\tau _{j}}(X(t))$ represent the lower and upper boundaries of the area covered by a pen of thickness $\tau _j$ at time *t*, respectively. As for the pen shape, Fryzlewicz & Oh (2011) considered square and round shapes as follows.

1. *Square pen*
  $$ U_{\tau }(X(t))=\max _{k\in [-\frac{\tau }{2}, \frac{\tau }{2}]\cap \mathbb {Z}} \Big \{X (t +k) \Big \} +\frac{\tau }{2}\gamma , $$
  $$ L_{\tau }(X(t))=\min _{k\in [-\frac{\tau }{2}, \frac{\tau }{2}]\cap \mathbb {Z}} \Big \{X (t +k) \Big \} - \frac{\tau }{2}\gamma . $$
2. *Round pen*
  $$ U_{\tau }(X(t))=\max _{k\in [-\frac{\tau }{2}, \frac{\tau }{2}]\cap \mathbb {Z}} \Big \{X({t+k})+\gamma \sqrt{\tau ^2/ 4-k^2}\Big \}, $$
  $$ L_{\tau }(X(t))=\min _{k\in [-\frac{\tau }{2}, \frac{\tau }{2}]\cap \mathbb {Z}} \Big \{X({t+k})-\gamma \sqrt{\tau ^2/ 4-k^2}\Big \}. $$

Above, $\mathbb {Z}$ denotes the set of integers, and $\gamma$ represents the scaling factor defined to adjust the difference between the thickness of the pen and the data variability. As Fryzlewicz & Oh (2011) suggested, $\gamma$ is always set to $\gamma =0.1$ unless otherwise stated.

**Fig. 4:**
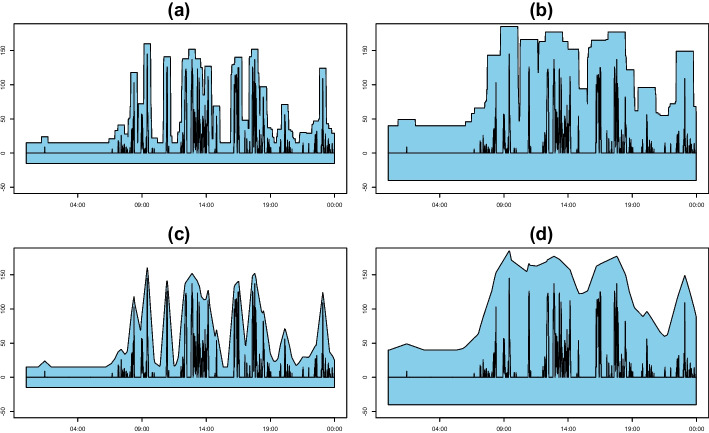
(a) and (b) TPT with thicknesses of 30 and 80. (c) and (d) e-TPT with thicknesses of 30 and 80. The original step data are plotted with a solid black line

The TPT has a multi-scale feature of viewing data at different distances according to the thickness of the pen. Specifically, applying large $\tau$ values corresponds to zooming out and coarsely viewing data trends, whereas small $\tau$ values sensitively capture the original features. Further, this transformation is visually intuitive and informative. Figure 4(a) and (b) display the boundaries obtained by applying a square pen with thicknesses of $\tau =30$ and 80, respectively, to the step data recorded on a specific day. The data trend with $\tau =80$ is coarser than that with $\tau =30$.

In this study, we consider a variation of the TPT to obtain a smooth version of the thick-pen boundaries, enhancing the temporal resolution of the time-series data and making the proposed clustering performance more effective. Thus, we define the upper and lower ensemble boundaries of a real-valued univariate process $\{ X(t)\}_{t=1}^T$ with a square pen with a thickness of $\tau$ as

$$ U^{\mathcal {E}}_{\tau }(X(t))=\frac{1}{\tau +1 }\sum _{\ell =0}^{\tau } {\max \Big \{X({t-\ell }),\dots ,X ( {t+\tau -\ell } ) \Big \}}+\frac{\tau }{2}\gamma , $$

$$ L^{\mathcal {E}}_{\tau }(X(t))=\frac{1}{\tau +1 }\sum _{\ell =0}^{\tau } {\min \Big \{X ({t-\ell }),\dots ,X({t+\tau -\ell }) \Big \}}-\frac{\tau }{2}\gamma , $$

which are the ensemble means of boundaries created with different starting points $\ell$s. Thus, the ensemble TPT (e-TPT) of $\{ X(t)\}_{t=1}^T$ is defined as the sequence of pairs of the ensemble boundaries,

$$ \textbf{TP}_\mathcal {J}^{\mathcal {E}}(X(t))=\Big \{ \Big ( L^{\mathcal {E}}_{\tau _j}(X(t)),U^{\mathcal {E}}_{\tau _j}(X(t)) \Big )\Big \}_{j=1,2,\ldots ,|\mathcal {J}|}. $$

Using the average value of the boundaries, the ensemble TPT provides smoother boundaries than the conventional TPT and is less sensitive to the initial data values and outliers. Figure 4(c) and (d) illustrate the ensemble boundaries with thicknesses of 30 and 80, where the boundaries are much smoother than the conventional ones in panels (a) and (b).

### Similarity Measure for Clustering

This section proposes a similarity measure employed as the input variable for clustering zero-inflated time-series data. For this purpose, we consider the thick-pen measure of association (TPMA) between the two time series *X*(*t*) and *Y*(*t*) proposed by Fryzlewicz & Oh (2011). Suppose that *X*(*t*) and *Y*(*t*) are on approximately the same scale. The TPMA is then defined as

1 $$\begin{aligned} \rho _{\tau }(X(t),Y(t)) \!=\! \frac{\min \{U_{\tau }(X(t)),U_{\tau }(Y(t))\}\!-\!\max \{L_{\tau }(X(t)),L_{\tau }(Y(t))\}}{\max \{U_{\tau }(X(t)),U_{\tau }(Y(t))\} \!-\! \min \{L_{\tau }(X(t)),L_{\tau }(Y(t))\}}, ~~ \text {for}~t \!=\!1, \ldots , T. \end{aligned}$$

Moreover, $\rho _{\tau }(X(t),Y(t)) \in (-1,1]$, and $\rho _\tau (X(t),Y(t)) >0$ holds when an overlap exists between the two boundaries, whereas $\rho _\tau (X(t),Y(t))<0$ when a gap exists between the two boundaries. This idea of measuring time series dependence based on the overlap or gap of pen areas is intuitively recognized through the visualization of transformations.

To reflect the characteristics of zero-inflated time series data, we propose a new similarity measure based on e-TPT and TPMA. From now on, we assume that the given time series data are nonnegative and zero-inflated. Then, the lower boundary of e-TPT for zero-inflated time series data rarely fluctuates. Therefore, it is natural to modify the TPMA measure of (1) to set the lower boundary of the pen to zero. Then, the modified TPMA measure, TPMA$_0$, is defined as

2 $$\begin{aligned} \eta _\tau ^{\mathcal {E}}(X(t),Y(t)) := \frac{\min \{U_\tau ^{\mathcal {E}}(X(t)),U_\tau ^{\mathcal {E}}(Y(t))\}}{\max \{U_\tau ^{\mathcal {E}}(X(t)),U_\tau ^{\mathcal {E}}(Y(t))\}}, \end{aligned}$$

for each time and its geometric mean over time has been proposed as a measure to assess the similarity between two time series. Here, $\eta _\tau ^{\mathcal {E}}(X(t),Y(t))$ measures the intersection length between $[0, U_\tau ^{\mathcal {E}}(X(t))]$ and $[0, U_\tau ^{\mathcal {E}}(Y(t))]$ as a proportion of the union’s size of these two intervals. Thus, $0 <\eta _\tau ^{\mathcal {E}}(X(t),Y(t)) \le 1$ holds for $\tau >0$. This measure returns a value close to 1 when the two time series are similar at time *t*. It is noticeable that the e-TPT transformation can affect the ratio due to the pen thickness. For example, the ratio is less affected when the pen is relatively thin, but the ratio can vary significantly when the pen is relatively thick compared to the data values.

**Fig. 5:**
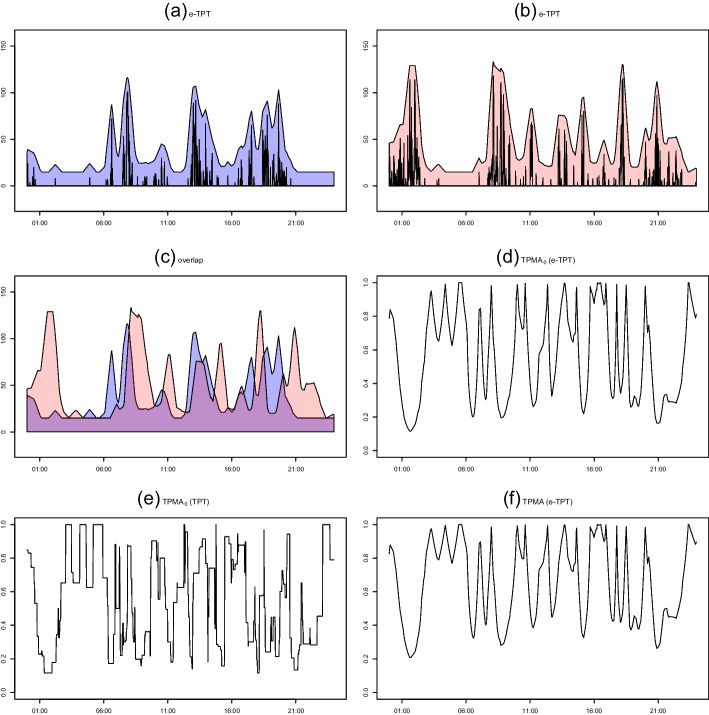
(a) and (b) e-TPT results using the square pen with a thickness of 30 for two step data. (c) Overlapping areas (purple) of the two e-TPT results. (d) TPMA$_0$ values based on e-TPTs, (e) TPMA$_0$ values based on TPTs. (f) TPMA values based on e-TPTs

Figure 5 presents the procedure for computing TPMA$_0$ for two step count time series. Panels (a) and (b) display the e-TPT results of the two dataset using a square pen with a thickness of 30, and panel (c) reveals the overlapping areas (purple) of the two e-TPT results. Finally, the result of the similarity measure $\eta _\tau ^{\mathcal {E}}(X(t),Y(t))$ of (2) is presented in panel (d). The measurement is low when a little overlap occurs between the two e-TPTs, whereas it is close to 1 when a considerable overlap exists. For comparison, we also present the TPMA$_0$ values based on TPT with a square pen in panel (e) and TPMA values based on e-TPT in panel (f). Both TPMA$_0$ and TPMA reflect the similarity between the two time series well. However, using (2), we can obtain more straightforward criteria for clustering, which is discussed in Section 2.3.

### Clustering Procedure Based on TPMA$_0$

The goal is to determine *K* optimal partitions of a set of observations $\textbf{X} = \{X_1, \dots , X_N\}$, where each $X_i$ belongs to a domain set *E*. We let $P = \{P_1, \dots , P_K\}$ be a set of *K* partitions of the data that satisfies $\bigcup _{c=1}^K {P_c} = \textbf{X}$ and $P_i \cap P_j = \varnothing$ for $i\ne j$. We set $M =\{ m_1, \dots , m_K : m_c \in E, c=1,\dots ,K \}$ as a set of cluster prototypes.

Given a distance function *d*, we define the clustering problem as minimizing the following cost function,

3 $$\begin{aligned} W(P,M) := \sum \limits _{c=1}^K { \sum \limits _{ X\in P_c} { d(X, m_c) } }. \end{aligned}$$

This optimization process is carried out in two steps using an iterative algorithm:

1. **Update** *P*: Given a set of cluster prototypes *M*, update *P* with
  $$ P_c = \{ X_i : \underset{m\in M}{\text {argmin}}~d(X_i,m) = m_c, ~i=1,\dots , N \} \text { ~~for each } c \in \{1,\dots ,K\}. $$
2. **Update** *M*: Given a partition *P*, update *M* with
  $$ m_c = \underset{m \in E}{\text {argmin}}\sum \limits _{X\in P_c} d(X,m) \text {~~ for each } c \in \{1,\dots ,K\}. $$

The cost function decreases for each iteration step. A well-known *K*-means algorithm (Hartigan & Wong, 1979) deals with $L_2$ distance, leading to the mean of each component as a cluster prototype when $E = \mathbb {R}^n,~n\in \mathbb {N}$. Furthermore, the $L_1$ distance function derives the *K*-medians algorithm using the medians as cluster prototypes (Leisch, 2006).

Suppose that we have multiple zero-inflated time series $X_i(t)$, $i=1,\ldots ,N$. We obtain the corresponding upper boundaries of $X_i(t)$ by e-TPT using a square pen with a thickness of $\tau$, $U_\tau ^{\mathcal {E}} (X_i(t))$, $i=1,\ldots ,N$. We compute the similarity measure TPMA$_0$ of (2) between any two time series data $X_i(t)$ and $X_j(t)$ ($i\ne j$) and take the $\log$ function. The measure can be further expressed as

$$\begin{aligned} \log \big (\eta _\tau ^{\mathcal {E}}(X_i(t),X_j(t))\big )= & {} \log \frac{\min \{U_\tau ^{\mathcal {E}}(X_i(t)),U_\tau ^{\mathcal {E}}(X_j(t))\}}{\max \{U_\tau ^{\mathcal {E}}(X_i(t)),U_\tau ^{\mathcal {E}}(X_j(t))\}}\\= & {} - \left| \log { \frac{U_\tau ^{\mathcal {E}} (X_i(t)) }{ U_\tau ^{\mathcal {E}} (X_j(t)) } } \right| . \end{aligned}$$

Given a partition $\{P_1, \ldots , P_K\}$, we let $c_i\in \{1,\ldots ,K\}$ be a cluster group label of the $X_i(t)$, and $m_{c_i}(t)$ be a cluster prototype of the group $X_i(t)$ belongs. Then, we maximize the geometric mean of the proposed similarity measure for each time *t* and element *i*, which is equivalent to minimize the sum of $L_1$ distance with respect to the logarithms of upper boundaries,

$$\begin{aligned} \underset{P, M}{\text {maximize}}~{\prod _{i=1}^N{\Big \{ \prod _{t=1}^{T}{\eta _\tau ^{\mathcal {E}}(X_i(t),m_{c_i}(t))}\Big \}^{1/T}}}\Longleftrightarrow & {} \! \!\underset{P, M}{\text {maximize}}~\sum \limits _{i=1}^{N}\sum \limits _{t=1}^T \frac{1}{T}\log \big ({\eta _\tau ^{\mathcal {E}}(X_i(t),m_{c_i}(t))}\big )\\\Longleftrightarrow & {} \! \!\underset{P, M}{\text {minimize}}~\!\sum \limits _{t=1}^T\!\sum \limits _{i=1}^{N}\left| \log U_\tau ^{\mathcal {E}}\!(X_i(t)) \!-\! \log U_\tau ^{\mathcal {E}}\!( m_{c_i} (t))\! \right| . \end{aligned}$$

In other words, given a partition and the cluster prototypes, we have the following cost function to be minimized,

4 $$\begin{aligned} W(P,M) =\sum \limits _{t=1}^T\sum \limits _{i=1}^{N}\left| \log U_\tau ^{\mathcal {E}}(X_i(t)) - \log U_\tau ^{\mathcal {E}}(m_{c_i}(t)) \right| . \end{aligned}$$

This problem is an $L_1$ optimization for the logarithms of the upper boundaries $\{\log U_\tau ^{\mathcal {E}}(X_i(t)),~i =1, \ldots , N\}$. Thus, applying the *K*-medians algorithm to this set ensures a monotonic decrease in the cost function.

As $0 <\eta _\tau ^{\mathcal {E}}(X_i(t),X_j(t))\le 1$ holds for $\tau > 0$, its log-transformation is problematic as $\eta _\tau ^{\mathcal {E}}(X_i,X_j )$ approaches zero. However, the thickness of a pen guarantees the a minimal value of the upper boundaries sufficiently greater than zero. Thus, we assume that $\delta$ exists such that $\eta _\tau ^{\mathcal {E}}(X_i(t),X_j(t))> \delta >0$ for any $i,~j \in \{1,\dots ,N\}$, as long as the upper boundaries of the transformed data are bounded above.

### Practical Algorithm

The entire clustering scheme can be summarized by Algorithm 1. Suppose that we have *N* zero-inflated nonnegative time series data, $X_1, \dots , X_N$. We assume that all time series data have the same scale, and the number of cluster groups *K* and the thickness $\tau$ are given. 
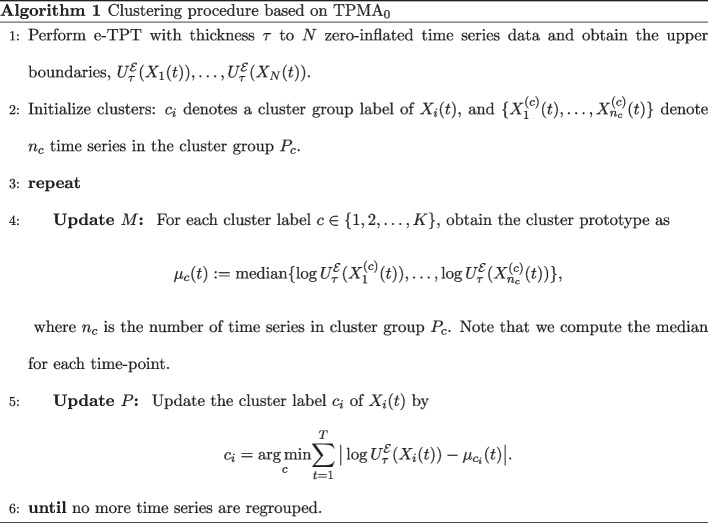


The followings are some remarks on the algorithm.

- Cost function (4) can be viewed as an $L_1$ optimization problem for the set of logarithms of upper boundaries $\{\log U_\tau ^{\mathcal {E}}(X_i(t)),~i =1, \ldots , N\}$. Therefore, we apply *K*-medians algorithm to this set and selecting the cluster prototype as the median of the logarithmic values as Step 4. It is worth noting that the corresponding $m_{c}(t)$, cluster prototype of $X \in P_c$ in (3), can be defined as the value satisfying $\mu _{c}(t) = \log U_\tau ^{\mathcal {E}}( m_{c}(t))$, which is not unique for each $\mu _{c}(t)$. However, the clustering algorithm works only with $\mu _{c}(t)$ and does not require to identify $m_{c}(t)$.
- This study considers various thickness values ($\tau$) for a multiscale interpretation of the results. Applying a thick pen tends to view data from a distance, focusing on significant trends; thus, the proposed clustering method divides the data based on global trends. Moreover, using a small value of thickness ($\tau$) tends to capture the pattern sensitively, and the corresponding clustering results reflect the detailed data pattern. However, in some cases, the optimal thickness ($\tau$) must be determined to obtain a single clustering result, where the cross-validation (CV) technique can be used to select the optimal value. More specifically, Algorithm 1 is applied to training data, and the cluster prototypes, $\mu _c(t)$, $c \in \{1,2, \ldots , K\}$ are obtained. Then, the cluster group label $c_i$ of test data $X^{te}_i$ is determined as
  $$ c_i = \underset{c}{\arg \min }\ { \sum _{t=1}^T{ { |\log { U^{\mathcal {E}}_{\tau } (X^{te}_i (t)) } -\mu _c (t) | } }}. $$
  The cross-validated error is defined as
  $$ CV = \frac{1}{n_{te}} \sum _{i=1}^{n_{te}} I ( c_i \ne c_{i,true}), $$
  where $n_{te}$ is the number of time series in the test data set, $c_{i,true}$ represents the true cluster group label of $X^{te}_i$, and *I* denotes the indicator function. A cluster validity index, such as the Dunn index (Pakhira et al., 2004) or Silhouette index (Shutaywi & Kachouie, 2021), may be used if the actual cluster groups are unknown.
- To determine the number of clusters *K*, we use the gap statistics from Tibshirani et al. (2001).

## Simulation Study

This section conducts a simulation study to evaluate the empirical performance of the proposed method. For this purpose, we consider four types of zero-inflated time series data. The true number of clusters, *K*, is assumed to be known in all cases. The reproducible R code for simulation studies is provided at https://github.com/mjkim1001/ZITS.

**Table 1:** Time-varying parameters in Model 1

Time varying parameters	Time index	Group $g=1$	Group $g=2$
$\phi _1^{(g)}$	$t =1, \ldots , 53$	0.8	0.8
	$t =54, \ldots , 128$	-0.9	1.6
	$t =129, \ldots , T$	0.8	0.8
$\phi _2^{(g)}$	$t =1, \ldots , T$	-0.81	-0.81

**Fig. 6:**
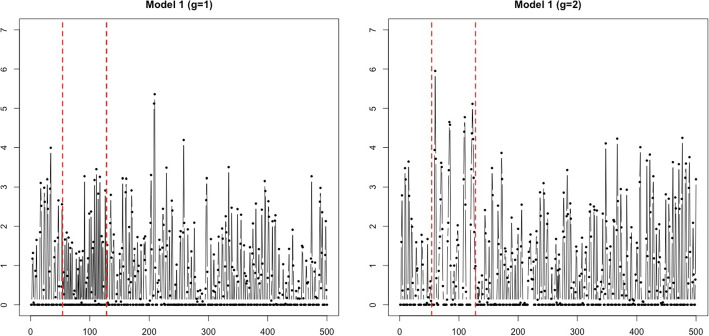
Sample time series with $T=500$ generated from Model 1. The red vertical lines indicate the change points, $t=54$ and 128

### Models for Simulation Data

**Model 1**: Nonstationary autoregressive model with abruptly changing parameters

This model was first considered by Fryzlewicz & Ombao (2009) for a classification problem. We modified the model slightly to have a zero-inflated time series structure and use it for clustering. The *i*th time series data from group *g*, denoted as $X^{(g)}_i(t)$, is generated from

5 $$\begin{aligned} X^{(g)}_i(t) = {\left\{ \begin{array}{ll} Y^{(g)}_i(t) ,&{} ~~\text {~~if~~} Y^{(g)}_i(t) \ge 0\\ 0 , &{} ~~~~~\text {otherwise, } \end{array}\right. } ~~~ \text {for}~~i=1, \ldots , N;~t=1, \ldots , T, \end{aligned}$$

where $Y^{(g)}_i(t) = \phi ^{(g)}_1 Y_i^{(g)} (t-1)+ \phi _2^{(g)} Y^{(g)} _i (t-2) + \epsilon ^{(g)} _i(t),$ and $\epsilon ^{(g)} _i(t)$ $\sim$ i.i.d. *N*(0, 1). The time-varying parameters $\phi _1^{(g)}$ and $\phi _2^{(g)}$ are defined as in Table 1, where $\phi _1^{(g)}$ are different at $t=54, \ldots , 128$. We generated $N=100$ time series from each group, and two sample time series with $T=500$ from each group are presented in Fig. 6. The average zero ratio of 100 time series is 0.501.

**Fig. 7:**
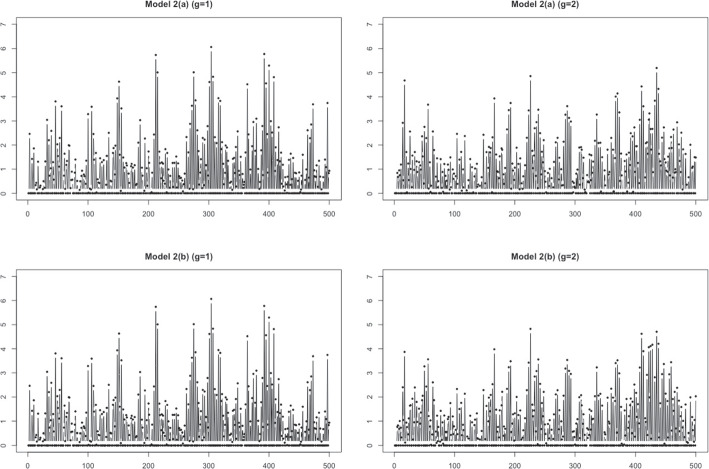
Sample time series generated from Model 2 with $T=500$

**Fig. 8:**
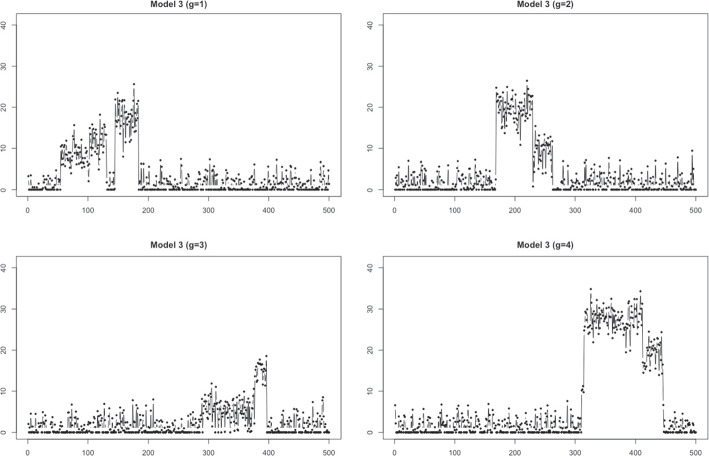
Sample time series generated from Model 3 with $T=500$

**Model 2** : Nonstationary AR model with slowly changing parameters

We generated two cases of data from a nonstationary AR model with slowly changing parameters. Thus, we used (5) with different $Y_{i}^{(g)}(t)$ forms: for $i=1, \ldots , N(=100);~t=1, \ldots , T(=500)$, and $\epsilon _{t}^{(g)}(t)$ i.i.d. *N*(0, 1) $(g=1,2)$,

**Fig. 9:**
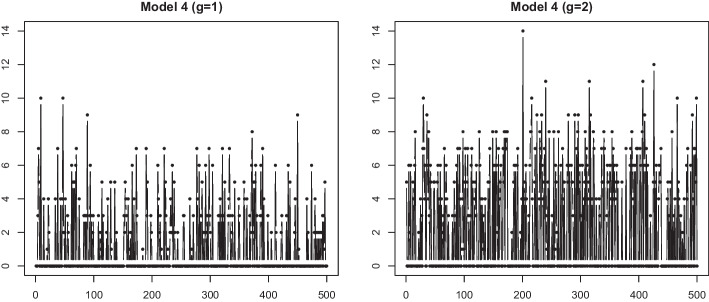
Sample time series generated from Model 4 with $T=500$ and $\sigma =0.1$

Case (a)

$$\begin{aligned} Y_{i}^{(1)}(t)= & {} - 0.8 [ 1- 0.7 \cos (\pi t/T)] Y_{i}^{(1)} (t-1) -0.81 Y_{i}^{(1)}(t-2) + \epsilon _{i}^{(1)}(t),\\ Y_{i}^{(2)}(t)= & {} - 0.8 [ 1- 0.001 \cos (\pi t/T)] Y_{i}^{(2)}(t-1) -0.81 Y_{i}^{(2)}(t-2) + \epsilon _{i}^{(2)}(t). \end{aligned}$$

Case (b)

$$\begin{aligned} Y_{i}^{(1)}(t)= & {} - 0.8 [ 1- 0.7 \cos (\pi t/T)] Y_{i}^{(1)} (t-1) -0.81 Y_{i}^{(1)}(t-2) + \epsilon _{i}^{(1)}(t)\\ Y_{i}^{(2)}(t)= & {} - 0.8 [ 1- 0.1 \cos (\pi t/T)] Y_{i}^{(2)}(t-1) -0.81 Y_{i}^{(2)}(t-2) + \epsilon _{i}^{(2)}(t). \end{aligned}$$

The average zero ratios for both cases are 0.5. The sample time series data from each group for both cases are illustrated in Fig. 7.

**Table 2:** Means and standard deviations (in parentheses) of the correct classification rate (CCR) and adjusted rand index (aRand) values

		Proposed TPT clustering			funFEM	funHDDC	DTW
		$\tau =20$	$\tau =30$	$\tau =50$			
Model 1							
($T=500$)	CCR	0.833(0.03)	0.847(0.029)	0.851(0.027)	0.853(0.027)	**0.871(0.028)**	0.842(0.133)
	aRand	0.445(0.079)	0.483(0.079)	0.493(0.076)	0.463(0.077)	**0.552(0.083)**	0.535(0.264)
($T=1000$)	CCR	0.812(0.064)	0.842(0.042)	**0.849(0.026)**	0.808(0.035)	0.824(0.036)	0.801(0.141)
	aRand	0.403(0.115)	0.473(0.090)	**0.488(0.072)**	0.383(0.087)	0.422(0.092)	0.439(0.264)
($T=1500$)	CCR	0.775(0.087)	0.820(0.074)	**0.844(0.029**)	0.798(0.034)	0.800(0.039)	0.670(0.145)
	aRand	0.329(0.150)	0.429(0.137)	**0.473(0.081)**	0.357(0.082)	0.364(0.092)	0.197(0.211)
Model 2							
(a)	CCR	**0.852(0.028)**	0.845(0.043)	0.84(0.026)	0.792(0.047)	0.791(0.044)	0.623(0.088)
	aRand	**0.496(0.078)**	0.480(0.091)	0.463(0.071)	0.347(0.110)	0.344(0.101)	0.089(0.102)
(b)	CCR	**0.827(0.029)**	0.826(0.029)	0.816(0.030)	0.758(0.045)	0.756(0.048)	0.593(0.076)
	aRand	**0.427(0.075)**	0.425(0.077)	0.400(0.076)	0.271(0.093)	0.268(0.098)	0.055(0.071)
Model 3							
	CCR	0.901(0.090)	0.893(0.098)	**0.905(0.063)**	0.821(0.142)	0.895(0.063)	0.526(0.067)
	aRand	0.781(0.152)	0.775(0.144)	**0.782(0.106)**	0.651(0.205)	0.762(0.107)	0.272(0.071)
Model 4							
($\sigma =0.1$)	CCR	**0.800(0.020)**	0.799(0.022)	0.798(0.023)	0.776 (0.021)	0.779(0.027)	0.763(0.034)
	aRand	**0.359(0.050)**	0.357(0.053)	0.355(0.055)	0.303(0.046)	0.311(0.06)	0.279(0.068)
($\sigma =0.5$)	CCR	**0.801(0.029)**	**0.801(0.029)**	0.799(0.029)	0.782(0.03)	0.778(0.036)	0.764(0.038)
	aRand	0.363(0.072)	**0.363(0.071)**	0.359(0.071)	0.319(0.068)	0.312(0.079)	0.281(0.088)

Bold face indicates the best performance

**Table 3:** Cross-validation results from each model

	Model 1			Model 2		Model 3	Model 4	
	$T=500$	$T=1000$	$T=1500$	(a)	(b)		$\sigma =0.1$	$\sigma =0.5$
Selected thickness	41.5 (20-100)	65(30-150)	74.22(20-150)	31(10-100)	37(20-100)	96(10-150)	42(10-150)	40(10-100)
CCR	0.853(0.027)	0.845(0.043)	0.837(0.053)	0.851(0.045)	0.828(0.038)	0.904(0.086)	0.804(0.021)	0.805(0.029)
aRand	0.499(0.077)	0.481(0.086)	0.463(0.107)	0.498(0.094)	0.433(0.089)	0.792(0.132)	0.370(0.051)	0.372(0.072)

Means and standard deviations (in parentheses) of the correct classification rate (CCR) and adjusted rand index (aRand) values, and means and ranges (in parentheses) of the selected thicknesses by CV

**Model 3** : Block data with different patterns

We considered a noisy block time series with four different patterns. To generate the time series, we reused (5) with the following $Y_{i}^{(g)}(t)$, $g\in \{1,2,3,4\}$ as

$$\begin{aligned} Y_i^{(g)}(t)= & {} \sum \limits _{j=1}^5{h_j\{ 1 + \text {sign}((t-1)/T - \xi ^{(g)}_j) \}/2} + \epsilon _i^{(g)}(t) ,~~\text {for}\\ i= & {} 1, \ldots , N(=100), ~t=1, \ldots , T(=500), \end{aligned}$$

where $\epsilon _i^{(g)} \sim N(0, 3^2)$, $g=1,2,3,4$. In addition, $h_j$ satisfies $|h_j|\sim U(0,20)$, $h_1, h_3 <0$, $h_2,h_4>0$, and $\sum _{j=1}^5 {h_j} = 0$, whose values are related to the height of each vertical jump, and $\xi ^{(g)}_j$ is generated from $U(\frac{g-1}{5} ,\frac{g+1}{5})$, for $g=1,2,3,4$. The average zero ratio of $N(=100)$ data from the above model is 0.494. The sample block time series data from each group are presented in Fig. 8.

**Model 4** : ZIP model with different mean

**Table 4:** Mean and standard deviations (in parentheses) computation times (sec) for a simulation

	Proposed TPT Clustering	funFEM	funHDDC	DTW
Model 1 ($T = 500$)	13.39 (2.54)	4.63 (1.02)	1.12 (0.72)	384.63 (39.41)

We considered a time series $X^{(g)}_i(t)$, $t=1, \ldots , T(=500)$, and $i=1,\ldots , N(=100)$, from group $g\in \{1,2\}$. The *i*th data in group *g* are generated from a zero-inflated Poisson model,

$$X_{i}^{(g)}(t) \sim ZIP (\lambda _{i}^g, \omega _i) ,$$

where $\lambda _{i}^g$ is the expected Poisson count generated from $N(\mu _i^{g}, \sigma ^2)$, $g=1,2$, $\mu _i^{1} \sim Unif(3,4)$ and $\mu _i^2 \sim Unif(2,10)$, and $\sigma =0.1,~0.5$. The zero-inflation parameter, $\omega _i$ is generated from *Unif*(0.4, 0.7). The average zero ratio from the generated data set is 0.583. Figure 9 displays the sample time series from two groups with $\sigma =0.1$.

**Fig. 10:**
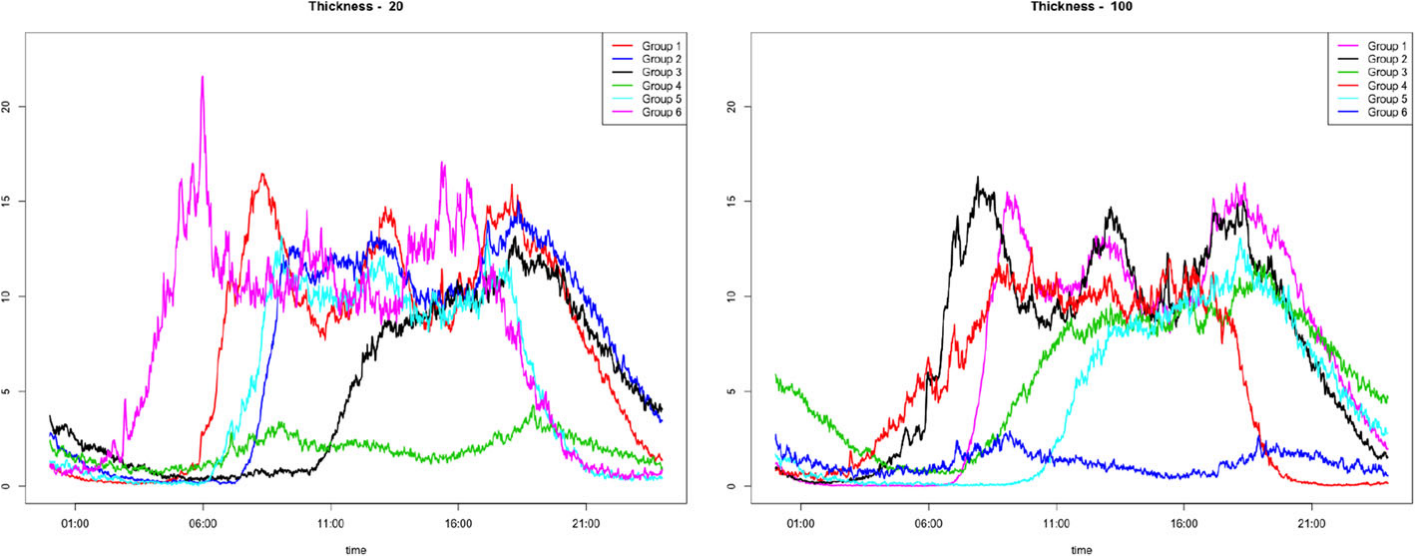
Mean time series of step data for each cluster by the proposed method with $\tau =20$ and 100

For comparison, we considered three existing functional and time series clustering methods:

- FunFEM – Functional clustering based on discriminative functional mixture modeling by Bouveyron et al. (2015). We use the default criterion in the R package “funFEM”.
- FunHDDC – Functional clustering based on the functional latent mixture modeling by Schmutz et al. (2018). We use the BIC to select the best model, and other hyper-parameters are set using default values in the R package “funHDDC”.
- DTW – Time-series clustering based on the dynamic time warping (DTW) distance by Wang et al. (2018), which is implemented using the R package “dtwclust” by Sarda-Espinosa (2022).

### Simulation Results

For the evaluation measure, we used the correct classification rate (CCR; %) and the adjusted Rand index (aRand) by Hubert & Arabie (1985). The aRand is a modified version of the Rand index (Rand, 1971), which adjusts the Rand index to have an expected value of 0 and to the upper bound of 1. It measures the correspondence between two partitions classifying the object pairs in a contingency table, and a higher value of the aRand index indicates a higher similarity between the two groups. Table 2 summarized the evaluation measures computed over 100 simulations.

In Model 1, the proposed TPT clustering with $\tau =50$ and funHDDC provides the best results. At $T=500$, funHDDC works best, but its performance rapidly decreases as *T* increases. The reduction in accuracy for large *T* is observed for all methods, but the proposed method with $\tau =50$ works well even for $T=1500$. For Model 2, the proposed methods outperform other clustering methods for Cases (a) and (b). The proposed method with $\tau =20$ provides the best results. We obtain similar results for Models 3 and Model 4. In particular, the proposed method with $\tau =50$ gives the best results in Model 3, and all proposed clustering results reveal similar performances in Model 4. The simulation results indicate that the proposed methods can improve accuracy compared to existing methods when an appropriate thickness is used. However, it should be noted that the performance of the proposed method relies on the choice of thickness and underlying model, which may be difficult to determine in practical applications. Overall, the proposed method generally utilizes a multiscale strategy for pen thickness to explore clustering results at various scales and demonstrates good clustering performance when selecting the appropriate pen thickness suitable for the data properties.

**Table 5:** Summary of clustering results obtained from the proposed method when $\tau =20$ and 100

		Cluster					
		1	2	3	4	5	6
	Number of Days	5865	4827	3471	3092	2710	1429
$\tau =20$	Mean Step Count	10817	10257	7283	2701	7441	11084
	Weekend (%)	10.6	31.7	45.8	38.3	30.7	11.5
	Number of Days	5380	5125	3704	2672	2543	1970
$\tau =100$	Mean Step Count	10124	10989	8416	7948	6264	1758
	Weekend (%)	22.3	9.0	44.8	23.8	47.0	39.2

**Fig. 11:**
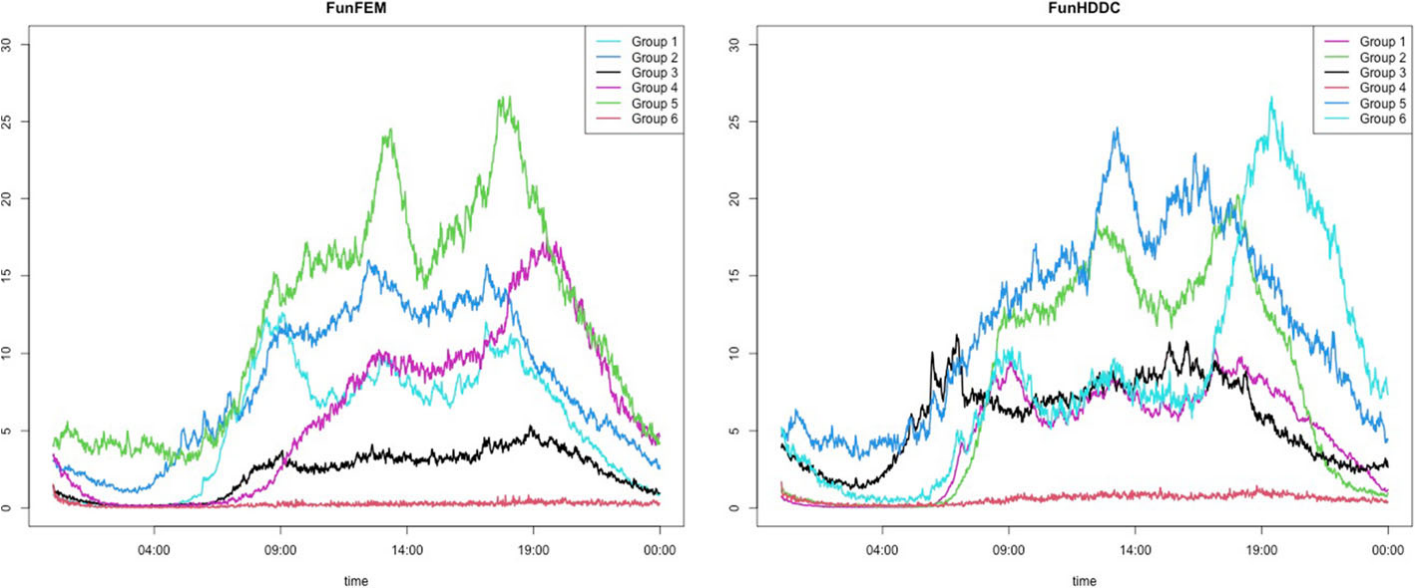
Mean time series of step data for each cluster by FunFEM and FunHDDC

As described in Section 2.4, we can use a five-fold CV to determine the optimal thickness for e-TPT. Table 3 summarizes the results. The CV results may perform worse than the proposed method with a specific thickness given in Table 2 in some cases, but they still offer better results than funFEM, funHDDC, and DTW, except Model 1 with $T=500$.

Finally, Table 4 summarized the computation time for each method conducted on the Model 1 dataset. For Model 1 with $T=500$, the proposed method took an average of 13.39 seconds to run a simulation on a desktop machine equipped with an Apple M1 Pro 8-core CPU and 16GB of memory. At the same setting, funFEM took 4.63 seconds, funHDDC took 1.12 seconds, and DTW took 384.63 seconds. The proposed method took longer than funFEM and funHDDC, but it only took around 22 minutes to run 100 simulations, which is a reasonable computation time compared to that of DTW.

## Real-data Analysis

### Step Count Data

We applied the proposed clustering algorithm to the step count data obtained from a *Fitbit*, a wearable device. The step data from 79 participants were measured every minute, and the number of recorded days varies from 32 to 364 per person. The total number of days in the dataset is 21,394. We first clustered the days based on patterns without considering inter- or intra- subject variability. The scaling parameter $\gamma$ is set to 0.2 for all cases, and the number of cluster groups is set to $K = 6$, which is determined by the gap statistic. Figure 10 illustrates the clustering results with the thicknesses $\tau =20$ and 100, and Table 5 lists the cluster size, mean step counts, and percentage of weekend days. Cluster groups are numbered in descending order, depending on the cluster size. For example, in the left panel of Fig. 10, Group 1 (red line) has the most number of days, and Group 6 (pink line) has the least number of days.

**Table 6:** Clustering validation measures

		Dunn index	VI
Proposed method	$\tau =20$	0.031	1.708
	$\tau =100$	0.023	1.726
FunFEM		0.018	0.877
FunHDDC		0.029	1.576

**Fig. 12:**
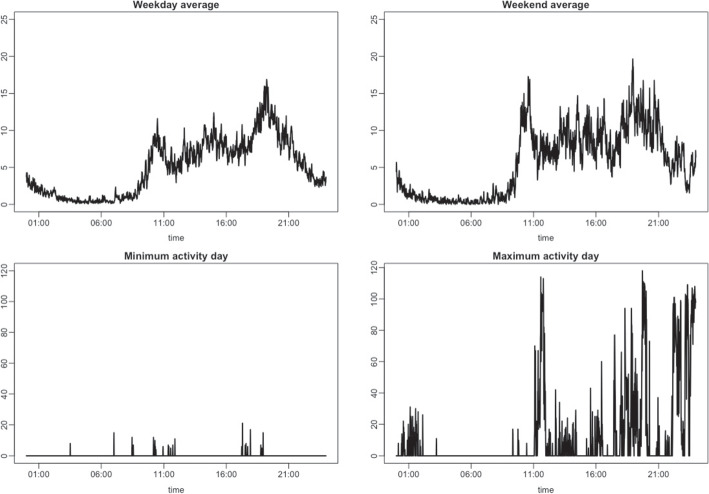
(Top) The average step data on weekdays and weekends of the 67th individual, and (Bottom) the step data from the least active day and the most active day

From the mean curves shown in the left panel of Fig. 10, it is noticeable that the proposed method classifies the pattern and amount of activities. Group 4 contains the least number of activity days, whereas Group 6 includes the most days. The time when the activity starts in Groups 1 and 6 is faster than in the other groups. In addition, in Table 5, we observe that these two groups contain more weekdays than weekends compared to other groups. When $\tau =100$, the mean time series in the right panel of Fig. 10 indicates that the proposed method properly classifies the days based on physical activity. The average pattern in each group is different from that in the left panel. For example, Group 3 contains days with activities that continued until midnight, and the days with this pattern are not grouped at $\tau =20$.

**Fig. 13:**
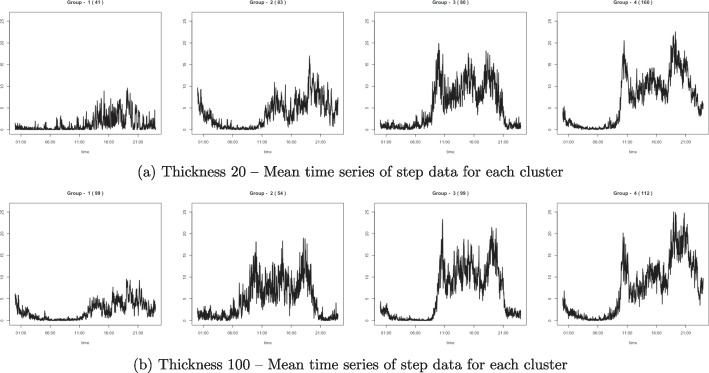
Clustering result of 67th participant by the proposed method: (a) Thickness 20 – Mean time series of step data for each cluster; (b) Thickness 100 – Mean time series of step data for each cluster

**Fig. 14:**
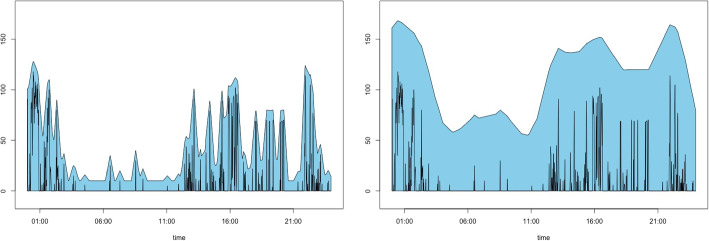
TPT when thickness is (left) 20 and (right) 100 for a selected day. The black solid line indicates the original step data

For comparison, the funFEM and funHDDC methods are applied to the step data. Figure 11 displays the mean time series of each group, which are different from the proposed method. The DTW clustering method is excluded because it took too long to compute the DTW distance between 21,394 time series.

The main difference between the clustering results using the proposed method and functional clustering methods is that the average number of steps in the least active group using the functional clustering methods is close to zero for all times, whereas the average time series of the least active group using the proposed method is far from zero. The proposed method uses the upper bound of e-TPT; thus, it is likely that time series with the most values of zero and those with all values of less than five are classified together using the proposed method. Depending on the purpose of the study, it may be essential to classify less-active days into one group. Therefore, the proposed clustering method can be used according to the purpose of the study.

We computed two clustering validation measures for the numerical validation of the clustering results: the Dunn index (Dunn , 1974) and variation of information (VI) (Meilă , 2007). The Dunn index measures the compactness of the intra-clusters and the inter-cluster separation, and VI measures the distance between clusters based on entropy. For both measures, a higher index indicates better clustering. In Table 6, the proposed method of $\tau =20$ and $\tau =100$ provides the highest values for the Dunn index and VI, respectively.

The proposed method can also be applied to cluster step data for a particular individual. For this purpose, we selected the 67th individual with 364 recorded days. To observe this individual’s activity patterns, we summarized their steps in Fig. 12, presenting the mean time series of the step data on weekdays and weekends and the data from the least and the most active days. We observe that, on weekends, activities continue until midnight compared to weekdays, and the activity level varies from day to day.

**Fig. 15:**
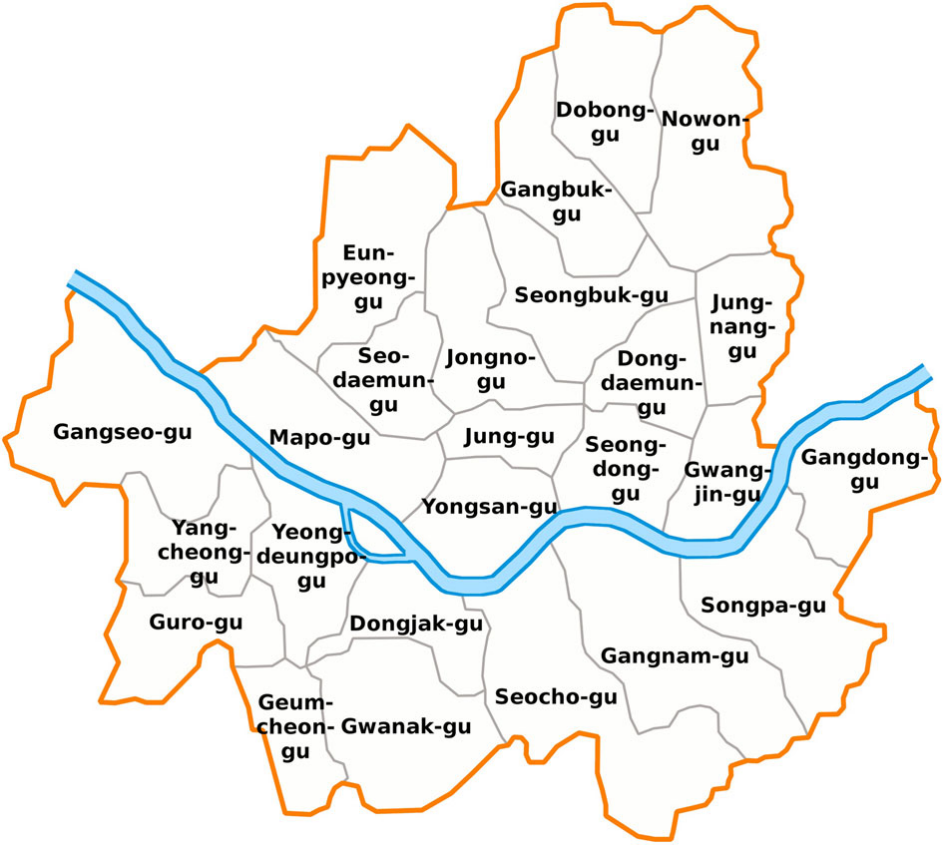
Districts of Seoul, Korea

The results of the proposed method are provided in Fig. 13. The average time series from the four groups represent various levels and patterns of activity. However, the results are slightly different depending on the pen thickness. Days with activity early at $\tau =20$ are clustered as Group 2 (moderate activity) but are not in one group when $\tau =100$. Moreover, e-TPT focuses on various aspects of the time series depending on the pen thickness. For example, we considered the day plotted in Fig. 14. When $\tau =20$, midnight activities are more evident, and the day is clustered as Group 2 in Fig. 13(a). However, the midnight activities do not seem to be much different from those in the morning when $\tau =100$, and we have relatively thick plots in the mornings, although there are few activities. The e-TPT is sensitive to the high activity intensity when using a thicker pen. Therefore, with $\tau =100$, the time series is clustered into the most active group: Group 4 in Fig. 13(b).

**Table 7:** The total number of newly confirmed COVID-19 cases, the number of days with zero confirmed cases, and the maximum confirmed cases per day from February 5, 2020, to June 18, 2021, according to district in Seoul, Korea

District	Total num of cases	Num of zero days (%)	Maximum confirmed cases in a day
Jongno-gu	791	258 (51.6%)	33
Jung-gu	741	250 (50.0%)	11
Yongsan-gu	1294	197 (39.4%)	28
Seongdong-gu	1296	201 (40.2%)	21
Gwangjin-gu	1570	218 (43.6%)	25
Dongdaemun-gu	1766	200 (40.0%)	32
Jungnang-gu	2091	192 (38.4%)	36
Seongbuk-gu	1957	183 (36.6%)	39
Gangbuk-gu	1370	226 (45.2%)	19
Dobong-gu	1467	187 (37.4%)	22
Nowon-gu	2175	175 (35.0%)	32
Eunpyeong-gu	2031	173 (34.6%)	31
Seodaemun-gu	1194	210 (42.0%)	15
Mapo-gu	1508	189 (37.8%)	31
Yangcheon-gu	1641	202 (40.4%)	33
Gangseo-gu	2273	158 (31.6%)	81
Guro-gu	1565	192 (38.4%)	40
Geumcheon-gu	794	249 (49.8 %)	14
Yeongdeungpo-gu	1756	180 (36.0%)	36
Dongjak-gu	1958	164 (32.8%)	36
Gwanak-gu	2197	131 (26.2%)	27
Seocho-gu	2058	159 (31.8%)	37
Gangnam-gu	2852	144 (28.8%)	38
Songpa-gu	2854	150 (30.0%)	29
Gangdong-gu	1900	183 (36.6%)	21

### Newly Confirmed COVID-19 Case Data

We considered the number of new COVID-19 cases per day in Seoul, Korea, from February 5, 2020, to June 18, 2021, as a time series of length 500. There are 25 districts in Seoul, as depicted in Fig. 15, and the total number of newly confirmed cases during this period is summarized in Table 7. The rates when the number of confirmed cases is zero during the given period are listed in the table and are higher than 28% in all districts. In Jongno-gu, there are zero confirmed cases on more than half of the days, and the highest number of confirmed cases, 81, is observed in Gangseo-gu.

**Fig. 16:**
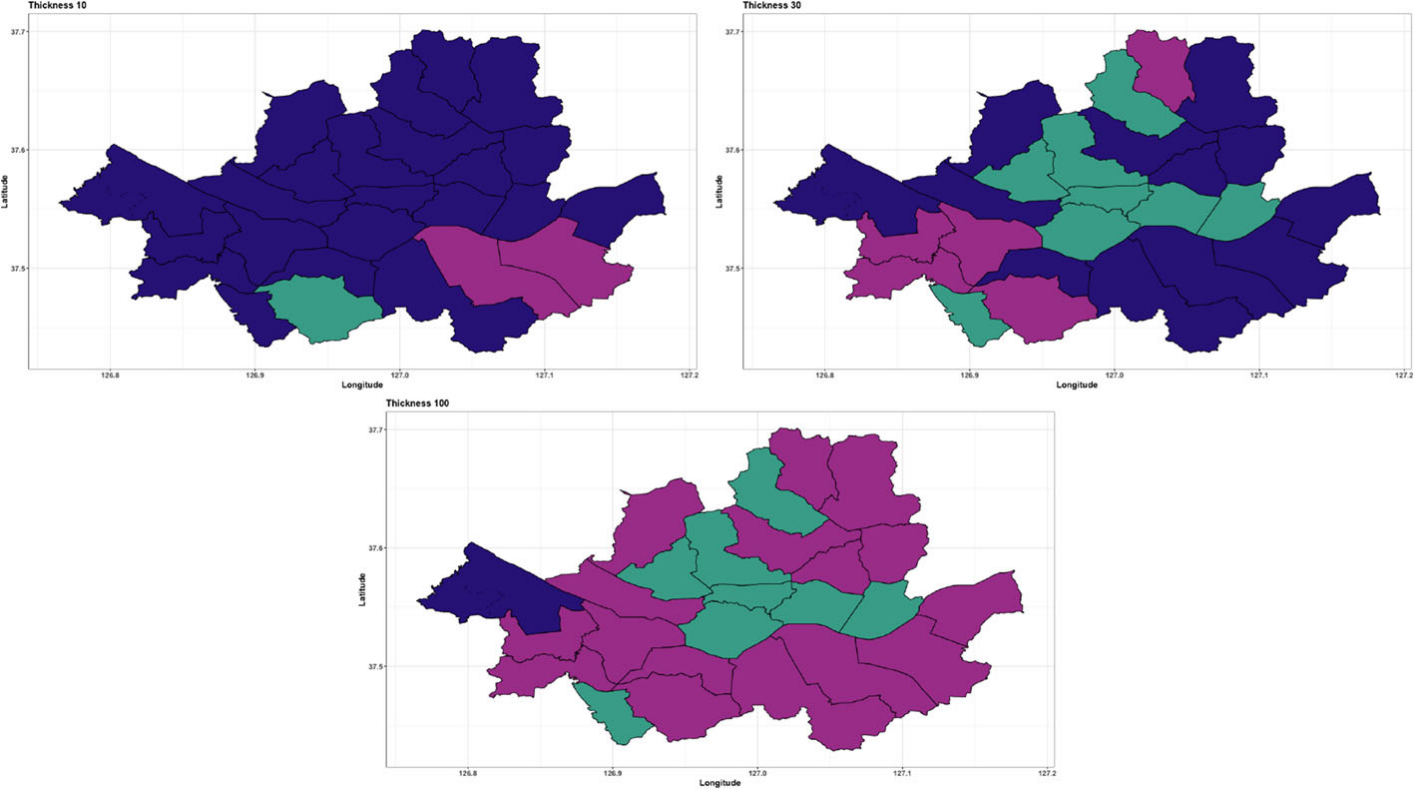
Clustering results by the proposed method when $\tau =10, 30$ and 100. Cluster groups are color-coded

**Fig. 17:**
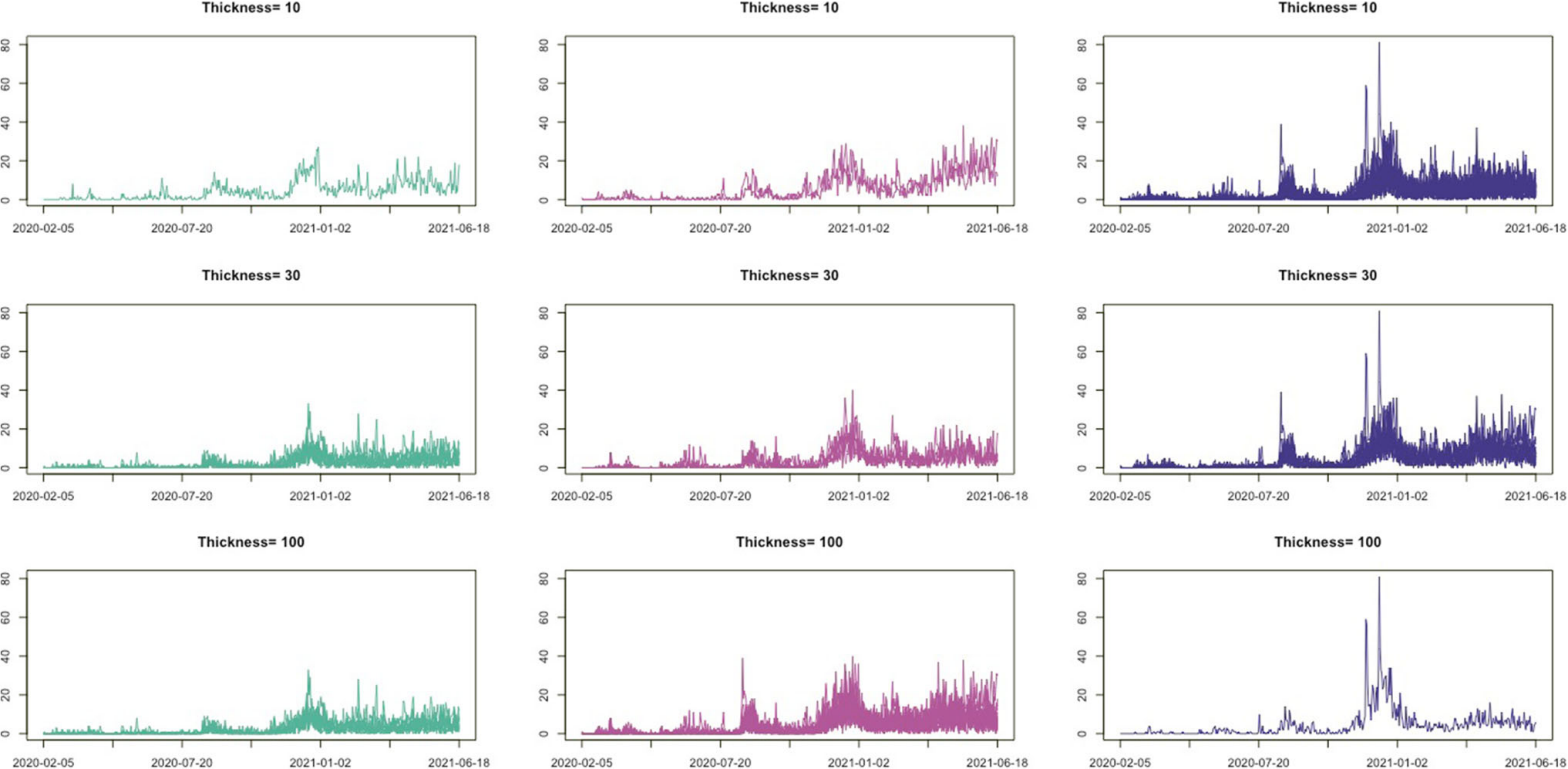
Mean time series of COVID-19 data for each cluster by the proposed method with $\tau =10, 30,$ and 100.

As illustrated in Fig. 3, the number of new COVID-19 cases per day in each district is zero-inflated time series data, and we apply the proposed method to cluster the 25 districts based on the time series patterns. The number of cluster groups is three, determined by the gap statistics. Figure 16 presents the clustering results using three different pen thicknesses ($\tau =$10, 30, and 100). The clustering results vary depending on the pen thickness. When $\tau =10$, only one district, Gwanak-gu, is classified as Group 1. Gwanak-gu has the least days with zero confirmed cases. Group 2 includes Gangnam-gu and Songpa-gu, and these districts have the two highest total confirmed cases during this period. When $\tau =100$, the proposed method brings out coarser-scale features of the data, and Gangseo-gu, which has the highest number of confirmed cases, is classified as a single group. Figure 17 displays the mean time series of each group, and Table 8 lists the summary statistics of the clustering results according to the pen thickness, such as the number of districts, average number of cases, and average rate of zero days. We observe that the levels and patterns of groups vary according to the pen thickness, and the statistics of the clustering results also vary.

**Fig. 18:**
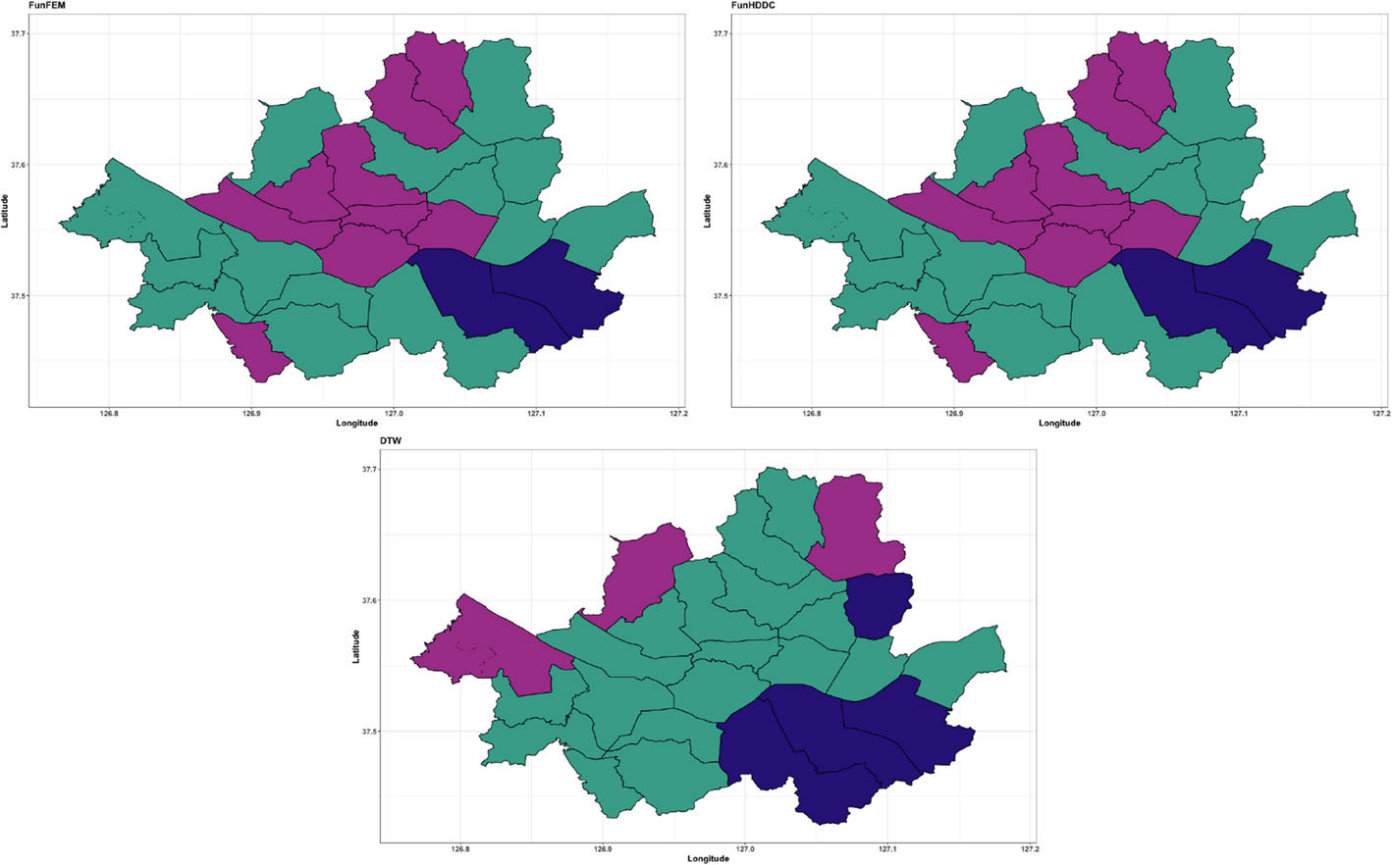
Clustering results obtained from FunFEM (top left), FunHDDC (top right), and DTW (bottom). Cluster groups are color-coded

We apply the FunFEM, FunHDDC, and DTW methods to compare the COVID-19 data. The clustering results are provided in Fig. 18. The results of FunFEM and FunHDDC are identical, and some parts are similar to those of the proposed method using a pen thickness of $\tau =100$. The Dunn index and VI for the clustering results are presented in Table 9. The proposed method with $\tau =10$ and DTW shows high Dunn indices, while the proposed method with $\tau =30$ yields the highest VI.

## Concluding Remarks

In this study, we proposed a novel clustering method that can be applied to high-dimensional zero-inflated time series data. By modifying the TPT, we developed the e-TPT to improve the temporal resolution of zero-inflated time series data and introduced a similarity measure for zero-inflated time series data as an input variable for the clustering algorithm. Furthermore, an efficient iterative clustering algorithm was proposed. Finally, the effectiveness of the proposed method was demonstrated using simulation experiments and real-data analyses with step count data and newly confirmed COVID-19 case data.

As e-TPT solves the problem of exceeding zero in zero-inflated data, the proposed method can cluster zero-inflated time series, which is commonly observed in various fields. In addition, the proposed method provides a multiscale view of the data by considering various thicknesses of the e-TPT. If we use a thick pen, we can cluster time series based on the global trend, and a thin pen renders cluster groups divided based on the local features of the data. Furthermore, the proposed method addresses missing data issues by utilizing the TPT, which can accommodate missing data through the consideration of a large thickness. Similarly, e-TPT also tackles missing data by transforming the raw series into smoothed time-series data.

**Table 8:** Summary statistics for each cluster obtained from proposed method with thicknesses of the pen 10, 30, and 100

Thickness		Cluster 1	Cluster 2	Cluster 3
10	Number of districts	1	2	22
	Average number of cases	4.394	5.706	3.200
	Average percentage of zero days (%)	26.20	29.40	39.51
30	Number of districts	8	5	12
	Average number of cases	2.263	3.450	4.237
	Average percentage of zero days (%)	45.23	35.68	34.50
100	Number of districts	8	16	1
	Average number of cases	2.263	3.972	4.546
	Average percentage of zero days (%)	45.23	35.05	31.60

**Table 9:** Clustering validation measures

		Dunn index	VI
Proposed method	$\tau =10$	0.534	0.443
	$\tau =30$	0.418	1.039
	$\tau =100$	0.469	0.779
FunFEM		0.361	0.895
FunHDDC		0.462	1.021
DTW		0.538	0.784

However, the time series length must be the same for the current algorithm to be applied. Future studies could explore to handle time series with varying lengths. Another issue in the proposed method is finding the pen’s optimal thickness. Although the CV technique has been used for the thickness selection in the current study, an optimal choice using a data-adaptive selection may improve the clustering performance of the proposed method. It is reserved for future research.

## Data Availability

Data available on request from the authors.
